# Transcriptome and metabolome profiling unveils the mechanisms of naphthalene acetic acid in promoting cordycepin synthesis in *Cordyceps militaris*

**DOI:** 10.3389/fnut.2023.1104446

**Published:** 2023-02-16

**Authors:** Xin Wang, Yin Li, Xiue Li, Lei Sun, Yetong Feng, Fangping Sa, Yupeng Ge, Shude Yang, Yu Liu, Weihuan Li, Xianhao Cheng

**Affiliations:** ^1^Shandong Key Laboratory of Edible Mushroom Technology, School of Agriculture, Ludong University, Yantai, China; ^2^Yantai Hospital of Traditional Chinese Medicine, Yantai, China; ^3^College of Plant Protection, Jilin Agricultural University, Changchun, China

**Keywords:** molecular mechanism, naphthalene acetic acid (NAA), metabolic pathway, regulatory network, transcriptome

## Abstract

Cordycepin, an important active substance in *Cordyceps militaris*, possesses antiviral and other beneficial activities. In addition, it has been reported to effectively promote the comprehensive treatment of COVID-19 and thus has become a research hotspot. The addition of naphthalene acetic acid (NAA) is known to significantly improve the yield of cordycepin; however, its related molecular mechanism remains unclear. We conducted a preliminary study on *C. militaris* with different concentrations of NAA. We found that treatment with different concentrations of NAA inhibited the growth of *C. militaris*, and an increase in its concentration significantly improved the cordycepin content. In addition, we conducted a transcriptome and metabolomics association analysis on *C. militaris* treated with NAA to understand the relevant metabolic pathway of cordycepin synthesis under NAA treatment and elucidate the relevant regulatory network of cordycepin synthesis. Weighted gene co-expression network analysis (WGCNA), transcriptome, and metabolome association analysis revealed that genes and metabolites encoding cordycepin synthesis in the purine metabolic pathway varied significantly with the concentration of NAA. Finally, we proposed a metabolic pathway by analyzing the relationship between gene–gene and gene–metabolite regulatory networks, including the interaction of cordycepin synthesis key genes; key metabolites; purine metabolism; TCA cycle; pentose phosphate pathway; alanine, aspartate, and glutamate metabolism; and histidine metabolism. In addition, we found the ABC transporter pathway to be significantly enriched. The ABC transporters are known to transport numerous amino acids, such as L-glutamate, and participate in the amino acid metabolism that affects the synthesis of cordycepin. Altogether, multiple channels work together to double the cordycepin yield, thereby providing an important reference for the molecular network relationship between the transcription and metabolism of cordycepin synthesis.

## 1. Introduction

*Cordyceps militaris*(*C. militaris*), also known as *North Cordyceps sinensis*, is an entomogenous fungus belonging to the Ascomycota, Hypocreales, and Ergotaceae. It is a model species of *Cordyceps* (1). The stromata of *C. militaris* is solitary or clustered, mainly growing from the head of the host, occasionally from the chest and abdomen of the insect. The color is mostly orange yellow, generally unbranched, 2 to 7 cm high. The stromata often develop into a cylindrical shape, occasionally oblate, wide in the middle, narrow at both ends, and rod-shaped (2). It has been reported that a variety of bioactive metabolites have been isolated from *C. militaris*, such as cordycepin, cordyceps polysaccharide, cordycepic acid (3), carotenoids, ergosterol and, cordyceps metallothionein, and pentostatin (4). Among these bioactive components, cordycepin has attracted considerable attention because of its antibacterial, anti-inflammatory, anti-tumor, anti-oxidation, and other biological functions (5–9). In addition, Rabie reported that cordycepin possesses antiviral activity and can effectively promote the comprehensive treatment of the new coronavirus COVID-19 disease (10). *C. militaris* was listed as a new resource food by the Ministry of Health in 2009 and approved as a new food raw material in 2014 and therefore has earned a broad market prospect (Announcement No. 3 of the Ministry of Health of the People’s Republic of China in 2009).

Cordycepin, as a secondary metabolite, its metabolic pathway and synthetic regulation are complex and have not been completely revealed. In terms of metabolic regulation of cordycepin synthesis, most previous studies used *C. militaris* mycelia as the research object and studied the selection of nutrients in the basic medium (11–13), the optimization of culture conditions (different light and temperature treatments) (14–16), and the addition of exogenous substances (amino acids, precursor of cordycepin synthesis, plant growth hormone, salt, vegetable oil) on cordycepin synthesis (11, 17–20). NAA is a plant growth regulator. It is a synthetic organic compound with physiological effects similar to those of the plant hormone IAA, and it regulates plant growth and development (21). Yuan et al. found that cordycepin content in *C. militaris* fruiting body was significantly increased by adding naphthylacetic acid (NAA) to *C. militaris* solid medium. In-depth study of the molecular mechanism of NAA affecting cordycepin metabolism is helpful to elucidate the metabolic synthesis pathway of cordycepin (22), and has guiding significance for the production of *C. militaris* mycelia and fruiting bodies with high cordycepin content.

The development of bioinformatics has resulted in the wide use of omics technology to study the synthesis of cordycepin and the involved metabolic pathways and processes. Researchers have successively predicted the possible metabolic pathway of its synthesis. Lin et al. reported that cordycepin uses adenosine as the precursor, first phosphorylating adenosine monophosphate (AMP) under the action of adenosine kinase (ADK), leading to the production of adenosine diphosphate (ADP) under the action of adenylate kinase (ADEK). Next, ADP generates 3’-deoxyadenosine diphosphate (3’-dADP) under the action of nucleotide reductase (RNRS), and 3’-dADP generates 3’-deoxyadenosine monophosphate by ADEK. Finally, cordycepin is synthesized by 5’-nucleotidase (23). Similarly, Xia et al. stated that cordycepin synthesis involved adenosine as the precursor and through a series of phosphorylations and dephosphorylations under the action of related enzymes produced cordycepin under the action of oxidoreductase. In addition, the metabolism of cordycepin was associated with the synthesis of pentostatin (24). Although Raethong et al. proposed a regulatory network involving adenosine, methionine, and cordycepin, they did not verify it at the molecular level. Thus, based on previous studies, we speculate that the synthesis of cordycepin involves a combined action of multiple metabolic pathways, except the nucleotide metabolic pathway (25).

To further reveal the related molecular mechanism for improved cordycepin content following NAA treatment, we conducted a joint analysis of transcriptome and metabolomics. The weighted gene co-expression network analysis (WGCNA) was used to identify gene modules with similar expression patterns, followed by conducting association analyses for cordycepin and adenosine content traits. Next, a gene regulatory network diagram was drawn to identify core genes in the module. Afterward, combined with the metabolomics data, the transcriptome and metabolomics association analysis was performed, and the gene–metabolite regulatory network was drawn. Further, the cordycepin synthesis metabolic pathway under NAA treatment was analyzed. Finally, a metabolism network sketch following the addition of NAA to cordycepin was drawn. In this study, related genes and metabolic pathways of cordycepin synthesis were discussed from the perspective of transcription and metabolism, providing a molecular basis for obtaining high cordycepin strain and breeding in the future.

## 2. Materials and methods

### 2.1. Strain and medium

Subject strains of wild *C. militaris* were collected from Mengshan Mountain, Mengyin County, Shandong Province. After isolation, purification, and domestication, the subject strain was obtained, numbered MS05, and stored in our laboratory. First, *C. militaris* strain was inoculated on the PDA solid medium for activation and cultured at 25°C in dark for 7 days. After activation, the spawn were inoculated into the PDA liquid medium with the inoculation amount of 1%, 140 r⋅min^–1^ and incubated at 25°C in the dark for 7 days for standby. Oat was used as the basic culture substrate of the fruiting body, 30 g in each bottle, and NAA solution was added in a gradient concentration of 0, 2,000, 3,000, 4,000, 4,500, and 5,000 mg/L, respectively. Afterward, all the media were sterilized in moist heat sterilization autoclave for 30 min at 121°C. After cooling, *C. militaris* liquid medium was inoculated into the oatmeal medium with an inoculation volume of 5 mL per bottle, and 20 replicates were made for each treatment. These were incubated at 25°C as dark culture for 12 days, and after scratching, these were placed under 200 lux, 20°C, and 95% humidity for mushroom production management, and collected when spores were ejected. All experiments were repeated thrice to ensure accuracy of the experiment.

### 2.2. Determination of *C. militaris* dry weight, cordycepin content, and residual NAA content in the fruiting body and oat culture medium

To determine the dry weight, we collected *C. militaris* fruiting body, the medium was cleaned, dried at 55°C to constant weight, and then weighed with an electronic balance. The cordycepin content was determined according to the method described for cordycepin products specified in the Agricultural Industry Standard of the People’s Republic of China (NY/T2116-2012). Residual NAA in the fruiting body and oat medium was determined according to three Memoirs of the Food and Drug Administration (26) No. 73.

### 2.3. Determination and analysis of transcriptome

Illumina Novaseq 6000 sequencing analysis was performed on five different NAA-treated *C. militaris* fruits with three biological replicates per treatment concentration. The total RNA was extracted using the TRIzol reagent kit (Invitrogen, Carlsbad, CA, USA) according to the manufacturer’s protocol. The quality of the RNA was assessed on an Agilent 2100 Bioanalyzer (Agilent Technologies, Palo Alto, CA, USA) and checked using RNase-free agarose gel electrophoresis. After the total RNA was extracted, eukaryotic mRNA was enriched by Oligo(dT) beads.

The enriched mRNA was fragmented into short fragments using a fragmentation buffer and reversely transcribed into cDNA with random primers. The purified double-stranded cDNA fragments were end repaired, A base added, and ligated to Illumina sequencing adapters. The ligation reaction was purified with the AMPure XP Beads (1.0X). Ligated fragments were subjected to size selection by agarose gel electrophoresis and polymerase chain reaction (PCR) amplified. The resulting cDNA library was sequenced using Illumina Novaseq6000 by Gene Denovo Biotechnology Co., (Guangzhou, China). Reads obtained from the sequencing machines include raw reads containing adapters or low-quality bases, which could affect the following assembly and analysis. Thus, to obtain high-quality clean reads, reads were further filtered by fastp (version 0.18.0) (27). The short reads alignment tool Bowtie2 (version 2.2.8) (28) was used to map reads to the ribosome RNA (rRNA) database. Next, the rRNA-mapped reads were removed. The remaining clean reads were further used for gene assembly and calculating gene abundance. An index of the reference genome was constructed and paired-end clean reads were mapped to the reference genome using HISAT2. 2.4 (29) with “-RNA-strandness RF” and other parameters set as a default. The mapped reads of each sample were assembled using StringTie v1.3.1 (30, 31) in a reference-based approach. For each transcription region, a FPKM (fragment per kilobase of transcript per million mapped reads) value was calculated to quantify its expression abundance and variations using the RSEM (32) software. RNA-seq data were deposited in the NCBI sequence Read Archive (SRA) under bioproject (PRJNA900787).

The R package was used for sample relationship analysis in this study. Based on the gene expression information, R^1^ was used to perform principal component analysis, and R and Pearson’s correlation coefficient were used to quantify the correlation between biological replicates. To identify differentially expressed genes in each group, RNA differential expression analysis was performed using the DESeq2 (33) software between two different groups [and by edgeR (34) between two samples]. The difference multiple was 1.5, and the error detection rate was a *P*-value less than 0.05.

### 2.4. Weighted gene co-expression network analysis

After filtering the low-expression genes, the gene expression value was imported into WGCNA, and the default automatic network building function blockwise modules were used to construct a co-expression module. The power value was 13, the similarity value was 0.7, and the minimum gene number of the module was 50. Genes were clustered into 18 related modules. The module eigenvalue was used to perform the association analysis with the cordycepin and adenosine content, identify the module most relevant to the cordycepin and adenosine content according to Pearson’s correlation coefficient, and afterward select the possible related genes with the cordycepin synthesis according to the relevant pathway enrichment analysis in the module. Cytoscape v.3.7.1 was used to draw the regulatory network interaction diagram of the internal genes of the module.

### 2.5. Untargeted metabolomics detection and data analysis group

Five *C. militaris* fruiting bodies were taken and treated with different concentrations of NAA, with each concentration having six biological replicates. The sample was transferred to an EP tube. After the addition of the extract solution (acetonitrile:methanol = 1:1, containing isotopically labeled internal standard mixture), the samples were vortexed for 30 s, sonicated for 10 min in an ice-water bath, and incubated for 1 h at −40°Cto precipitate proteins. Next, the sample was centrifuged at 12,000 rpm for 15 min at 4°C. The resulting supernatant was transferred to a fresh 2 mL LC/MS glass vial for the UHPLC-QE-MS analysis. The quality control (QC) sample was prepared by mixing an equal aliquot of supernatants from all samples. LC-MS/MS analyses were performed using an UHPLC system (Vanquish, Thermo Fisher Scientific) with a UPLC BEH Amide column coupled to a Q Executive HFX mass spectrometer (Orbitrap MS, Thermo). The QE HFX mass spectrometer was used for its ability to acquire the MS/MS spectra on information-dependent acquisition (IDA) mode in the acquisition software (Xcalibur, Thermo). In this mode, the acquisition software continuously evaluated the full-scan MS spectrum. The raw data were converted to the mzXML format using ProteoWizard and processed with an in-house program, which was developed using R and based on XCMS, for peak detection, extraction, alignment, and integration. Next, an in-house MS2 database (BiotreeDB) was applied for metabolite annotation. The PCA analysis of unsupervised patterns was conducted using R-packet GModels. In addition, partial least square discriminant analysis (PLS-DA) was performed, which is a multivariate statistical method with supervised pattern recognition. Orthogonal least partial square discriminant analysis (OPLS-DA) was derived from PLS-DA. Compared with PLS-DA, OPLS-DA is a combination of orthogonal signal correction (OSC) and PLS-DA. The data obtained from metabolite analysis were standardized for PCA and OPLS-DA. A combination of variable importance and *t*-test in the projection (VIP) score of the OPLS model was used to determine differential cumulative metabolites (DAMs). Those with *p*-values, *t*-tests < 0.05 and VIP ≥ 1 were considered differentially abundant metabolites (DAMs) between the two groups. Pathway significance enrichment analysis considers the KEGG pathway as a unit and applies the hypergeometric test to identify the pathways significantly enriched in DAMs compared with the background genes. The *p*-value of this hypothesis test after the correction was 0.05 as the threshold, and the eligible pathway was defined as the one significantly enriched in DAMs.

### 2.6. Transcriptome and metabolome correlation network

Based on the expression quantity and metabolite abundance, the functional model of the pathway was analyzed to identify the KEGG metabolic pathway shared by genes and metabolites. Subsequently, the association characteristics between genes and metabolites in the shared pathway were analyzed. The Pearson correlation coefficient was calculated according to the transcriptome gene expression (FPKM), and the relative content of metabolite to obtain the correlation between metabolomics and transcriptome data. The pairs of genes and metabolites were arranged in the descending order of absolute correlation coefficient.

### 2.7. Real-time quantitative polymerase chain reaction (RT-qPCR)

The same RNA samples used in RNA-Seq were used for RT-qPCR. According to the instructions of the reverse transcription kit (R223; Vazyme Biotech, Nanjing, China), a 20 μL reaction system was established with 50 ng to 2 μg of the total RNA, incubated at 50°C for 50 min, and incubated at 85°C for 5 min to obtain the cDNA. Next, the cDNA was loaded into the 96-well plate, and StepOnePlus (ABI, CA, USA) and RT-PCR reagent (Q341; Vazyme Biotech, Nanjing, China) were used for the qRT-PCR analysis. The 20 μL reaction system consisted of 10 μL 2 × ChamQ SYBR qPCR Master Mix (Vazyme Biotech, China), 0.4 μL PCR forward primer (10 μM), 0.4 μL PCR reverse primer (10 μM), 4 μL cDNA template and 5.2 μL ddH_2_O. PCR conditions are as follows: 95°C for 90 s, 95°C for 5 s, 60°C 15 s, and 72°C for 20 s, with a total of 40 cycles. The relative gene expression was analyzed according to the 2^–Δ^ *^Ct^* method. The product specificity and reaction efficiency of each primer pair were verified.

## 3. Results

### 3.1. Growth characteristics, fruiting body dry weight, and cordycepin content of *C. militaris* after NAA treatment

**FIGURE 1:**
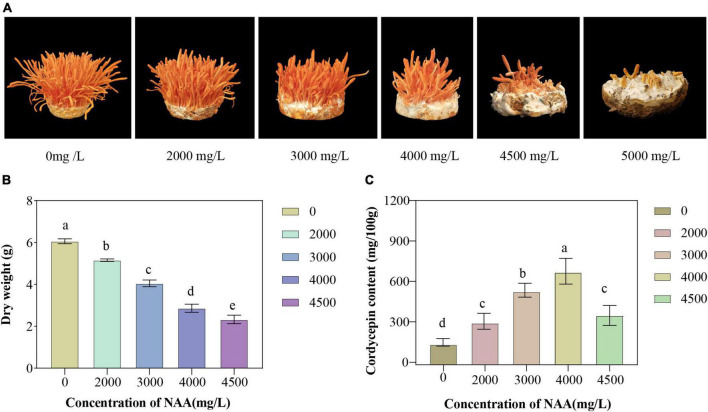
Growth characteristics, fruiting body dry weight, and cordycepin content of *Cordyceps militaris* after naphthalene acetic acid (NAA) treatment. **(A)** Growth morphology of *C. militaris* fruiting body treated with different concentrations of NAA. **(B)** Effects of different concentrations of NAA on *C. militaris* fruiting body dry weight. **(C)** Effects of different concentrations of NAA on cordycepin content. Data were analyzed with Duncan’s multiple range test. Different letters indicate significant differences (*P* < 0.05).

The yield and cordycepin content of *C. militaris* treated with different concentrations of NAA were significantly different from those of the control group (Figure 1). The addition of NAA inhibited the growth of the fruiting body of *C. militaris*. With an increase in the NAA concentration, the dry weight of *C. militaris* decreased significantly, especially at 4,500 mg/L, the growth of *C. militaris* was significantly inhibited. When the concentration exceeded 5,000 mg/L, *C. militaris* could not grow (Figure 1A). The *C. militaris* fruiting body dry weight was measured. According to the results, the dry weight decreased with an increase of the NAA content. At 4,500 mg/L, the dry weight of *C. militaris* was the lowest (Figure 1B). In addition, *C. militaris* treated with different NAA concentrations were determined by high-performance liquid chromatography. With an increase in the NAA concentration, the content of cordycepin increased first, followed by a decrease. At 4,000 mg/L, the cordycepin content reached the maximum, i.e., 675.9 mg/100 g, which was 4.6 times that of the control group (Figure 1C). Results of the remaining NAA content in the *C. militaris* fruiting body and oat medium showed that the amount of NAA detected in the *C. militaris* fruiting body and germ was considerably less than the added content, and the NAA content in the fruiting body was substantially less than the oat medium (Table 1).

**TABLE 1:** Detected amount of naphthalene acetic acid (NAA) in *Cordyceps militaris* fruiting body and oat medium.

NAA addition concentration (mg/L)	NAA detected in fruiting bodies (mg/kg)	The amount of NAA detected in oat medium (mg/kg)
0	—	—
2000	4.7	7.6
3000	5.2	9.1
4000	6.1	14
4500	6.6	24

### 3.2. Transcriptome differential analysis of cordycepin synthesis related genes in response to NAA

**FIGURE 2:**
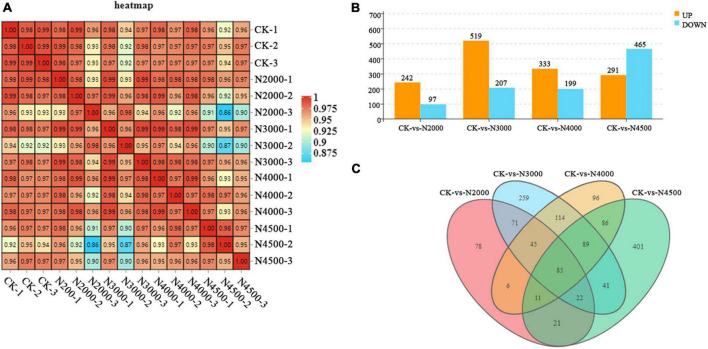
Overall qualitative and quantitative analyses of the transcriptome data. **(A)** A heatmap with pearson correlation analysis of control and different treatment samples. **(B)** Histogram of DEGs of different samples. **(C)** Venn diagram of control and different samples.

Transcriptome sequencing was performed on *C. militaris* fruiting body under different treatments with different concentrations of NAA. A total of 94 G of data were obtained. After removing the connectors and low-quality reads, we obtained a higher level of clean reads, with Q30 > 93% and more than 88% of the mapped reads of the reference genome, ensuring the reliability of the subsequent analysis (Supplementary Table 1). A heatmap with Pearson’s correlation coefficient analysis showed that the correlation coefficients between the samples were all greater than 0.86, showing a good correlation (Figure 2A). PCA revealed that the contribution rate of the first two principal components was 72.9%. The control group displayed distinct clusters with samples of 3,000, 4,000, and 4,500 mg/L concentrations, which were distinguishable. However, these partially overlapped with the concentration of 2,000 mg/L. However, the overall trend of groups was consistent with the PCA results of metabolomics samples (Supplementary Figure 1). In this study, *P* < 0.05 and a difference multiple of 1.5 were used as the threshold to screen DEGs. The 2,000, 3,000, 4,000, and 4,500 mg/L groups had 339 (242 upregulated and 97 downregulated), 726 (519 upregulated ones and 207 downregulated ones), 532 (333 upregulated ones and 199 downregulated), and 756 (291 upregulated ones and 465 ones) DEGs, respectively (Figure 2B). With an increase in the concentration, the number of upregulated DEGs first increased and then decreased, and reached the maximum at a concentration was 3,000 mg/L. The number of downregulated genes first increased and then decreased, and reached the maximum at a concentration of 4,500 mg/L. After treatment with different concentrations of NAA, the number of genes expressing significant changes first increased, followed by a decrease and then an increase. Compared with the control group, 1,425 DEGs were detected with 2,000, 3,000, 4,000, and 4,500 mg/L experimental groups, and 85 DEGs were identified between the control and other experimental groups; 78, 259, 96, and 401 DEGs were expressed in experimental groups (Figure 2C).

### 3.3. Screening of hub genes related to cordycepin synthesis

**FIGURE 3:**
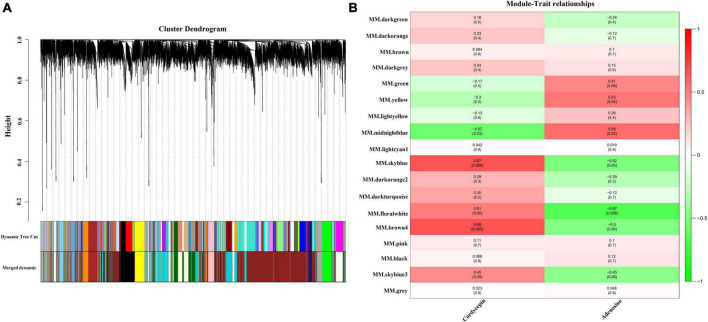
Weighted gene co-expression network analysis (WGCNA) of the significantly changed transcripts. **(A)** Module level clustering diagram, divided into 18 modules **(B)** cordycepin and adenosine content correlation diagram.

The WGCNA gene-weighted co-expression network analysis was used to construct a regulatory network with cordycepin synthesis-related genes. After the merging of the hierarchical clustering of modules, the clusters were clustered into 18 modules (Figure 3A), and afterward, the association analysis of these modules with cordycepin and adenosine content was conducted. The results demonstrated that cordycepin and adenosine were negatively correlated. Brown 4 module had the highest correlation with cordycepin content (*p* = 0.005, *r* = 0.68), followed by sky blue module (*p* = 0.006, *r* = 0.67), and midnight blue module showed a negative correlation with cordycepin content (*p* = 0.03, *r* = −0.57) (Figure 3B). Moreover, the highest module associated with the adenosine content correlation coefficient was the midnight blue module (*p* = 0.02, *r* = 0.58), followed by the yellow module (*p* = 0.04, *r* = 0.53), and the negative correlation module was the floral white (*p* = 0.006, *r* = −0.67).

To determine the dynamic changing trend of genes in each module, KEGG enrichment analysis was conducted to understand different biological change processes. Brown 4 module showed eight pathways that were significantly enriched with cordycepin content: Peroxisome, glycerolipid metabolism, ABC transporters, valine, leucine and isoleucine biosynthesis, longevity-regulating pathway multiple species, protein processing in the endoplasmic reticulum, 2-oxocarboxylic acid metabolism, pantothenate, and CoA biosynthesis. Supplementary Table 2 shows the enrichment of the Brown 4 module and other modules.

**FIGURE 4:**
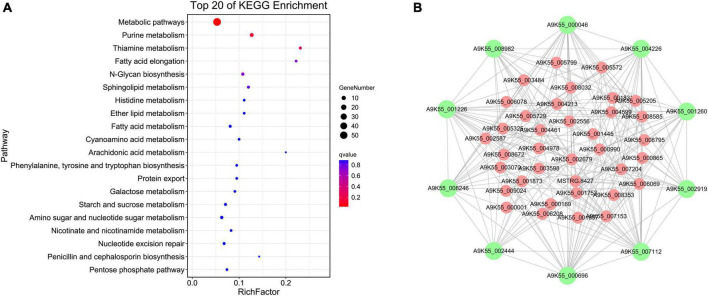
Co-expression network analysis of floral white module. **(A)** KEGG enrichment bubble diagram for floral white module. **(B)** Regulation of the floral white module gene network.

The floral white module revealed four significantly enriched pathways of cordycepin content: purine metabolic pathway, thiamine metabolic pathway, fatty acid elongation, and N-glycan biosynthesis (Figure 4A, Other pathway enrichments for this module are shown in Supplementary Table 3). Compared with the brown 4 module, the genes related to the cordycepin synthesis accounted for a relatively large proportion of the total number of genes in the module. Therefore, the floral white module was primarily analyzed, and a regulatory network of genes was constructed to identify hub genes. The floral white module network is shown in Figure 4B, the top 10 central genes were represented by large green circles; among these, A9K55_002919 was assigned to the purine metabolic pathway, A9K55_000696 was assigned to the sphingolipid metabolic pathway, A9K55_002444 was assigned to the lipid metabolic pathway, A9K55_001226 and A9K55_001260 genes were assigned to phosphoinositide metabolism and phosphoinositide metabolic pathway, A9K55_000046 was assigned to histidine metabolism, A9K55_007112 was assigned to the starch and sucrose metabolism and cyanoamino acid metabolism, A9K55_004226 was assigned to glycine, serine, and threonine metabolism, A9K55_008246 was assigned to ABC transporters, A9K55_008982 was assigned to different types of n-glycan biosynthesis pathways(Supplementary Table 4), suggesting that hub genes may were assigned to other metabolic pathways in addition to the purine metabolic pathway.

### 3.4. Differential analysis of metabolite response to NAA in *C. militaris*

**FIGURE 5:**
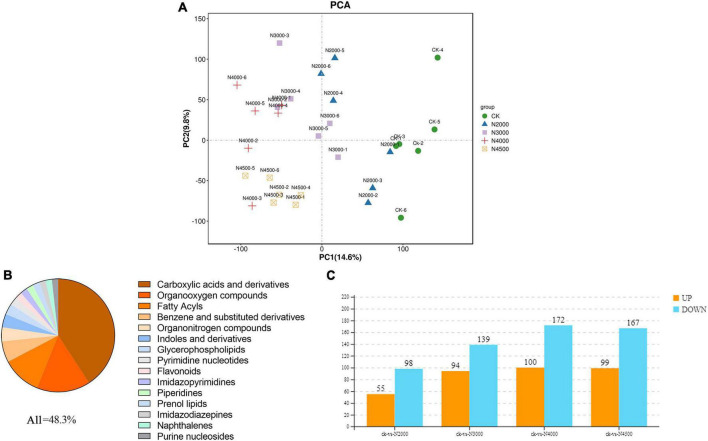
The proportion of differentially abundant metabolites (DAMs) identified by the metabolome is shown in the figure for the top 15 metabolite categories. **(A)** PCA analysis of the control group and four samples with different concentrations **(B)** of differentially abundant metabolites (DAMs) identified by the metabolome is shown in the figure for the top 15 metabolite categories. **(C)** Histogram of different metabolites between control and different treatment groups.

The dynamic changes in metabolites in five different concentrations of *C. militaris* were evaluated by LC-MS. We performed qualification control and repeatability analysis to show the stability of instruments and ensured the reliability and repeatability of the metabonomic data. The PCA and pearson correlation coefficient analysis results showed that experimental and control groups were clustered into different clusters, with evident differences (Figure 5A, Supplementary Figure 2). In this case, OPLS-DA showed separation between any two comparison groups. Therefore, the above multivariate statistical analysis results showed that these data have good repeatability and credibility and can be used for subsequent analysis (Supplementary Figure 3).

The VIP value of multivariate statistical analysis OPLS-DA and the *p*-value of univariate statistical analysis *t*-test were used to screen DAMs among different comparison groups. The threshold value of the difference was VIP ≥ 1 and *t*-test *p* < 0.05 in the OPLS-DA model. In total, 924 DAMs (Supplementary Table 5) were determined by control and experimental groups, of which 41% were not noted, and the other top 15 metabolite categories were carboxylic acids and derivatives (20%), organo-oxygen compounds (7.4%), fatty acyls (5.4%), benzene and substituted derivatives (2.9%), organonitrogen compounds (1.95%), indoles and derivatives (1.84%), glycerophospholipids (1.52%), pyrimidine nucleotides (1.08%), flavonoids (0.97%), imidazopyrimidine (0.97%), piperidines (0.97%), prenol lipids (0.97%), imidazodiazepine (0.86%), naphthalene (0.86%), and purine nucleosides (0.86%) (Figure 5B). The 2,000 mg/L groups had 153 DAMs (55 upregulated ones and 98 downregulated ones), and most of them were carboxylic acids and derivatives, fatty acyls, organooxygen compounds; in addition, piperidines and organonitrogen compounds were up-regulated, pyrimidine nucleotides, benzene, and substituted derivatives were down-regulated. The 3,000 mg/L groups had 233 DAMs (94 upregulated ones and 139 downregulated ones), and most of them were carboxylic acids and derivatives, fatty acyls, and organooxygen compounds; organonitrogen compounds, benzene, and substituted derivatives were also up-regulated, glycerophospholipids, benzene and substituted derivatives were down-regulated. The 4,000 mg/L group had 272 DAMs (100 DAM upregulated ones and 172 downregulated ones), and most of them were carboxylic acids and derivatives, fatty acyls, organooxygen compounds, benzene and substituted derivatives; purine nucleosides, keto acids and derivatives were also up-regulated, glycerophospholipids, indoles and derivatives, pyridines and derivatives were down-regulated. The 4,500 mg/L group had 266 DAMs (99 upregulated ones and 167 downregulated ones), and most of them were carboxylic acids and derivatives, fatty acyls, organooxygen compounds, benzene and substituted derivatives; hydroxy acids and derivatives, and organonitrogen compound were up-regulated, indoles and derivatives and glycerophospholipids were down-regulated (Figure 5C). Moreover, hierarchical cluster analysis evaluated the DAM accumulation pattern in different groups (Supplementary Figure 4).

To study the functions of metabolite after different NAA processing, the KEGG database was used to annotate the functions of the control group and the 2,000 mg/L group, the control group and the 3,000 mg/L group, the control group and the 4,000 mg/L group, the control group and the 4,500 mg/L group. In the control and 2,000 mg/L groups, galactose metabolism and lysine degradation pathway were significantly enriched. In the control and the 3,000 mg/L groups, ABC transporters, nitrogen metabolism, and lysine degradation pathway were significantly enriched. In the control and 4,000 mg/L groups, biosynthesis of amino acids, aminoacyl-tRNA biosynthesis, 2-oxocarboxylic acid metabolism, nitrogen metabolism, valine, and leucine and isoleucine biosynthesis pathway were significantly enriched. In the control group and the 4,500 mg/L group, nitrogen metabolism, ABC transporters, C5-branched dibasic acid metabolism, carbapenem biosynthesis, 2-oxocarboxylic acid metabolism, aminoacyl-tRNA biosynthesis, lysine degradation, glutathione metabolism, biosynthesis of secondary metabolites, biosynthesis of antibiotics, biosynthesis of amino acids, glyoxylate and dicarboxylate metabolic pathways pathway were significantly enriched (Supplementary Figure 5).

### 3.5. Regulation relationship between cordycepin synthesis-related genes and metabolite

**FIGURE 6:**
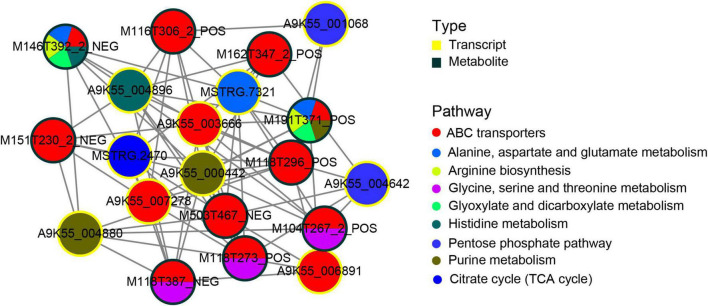
Transcript-metabolite Pearson’s correlation network representing differentially abundant metabolites (DAMs) and DEGs involved in *C. militaris*. Gene–metabolite pairs are connected by edges within the network. The yellow nodes represent genes and the green nodes represent metabolites. Edges between nodes represent correlations. The top 205 relationship pairs with correlation coefficient values were included in the network, and only the top 10 genes and top 10 metabolites are shown in the figure. The pie chart on the node shows a part of the pathways involved in DAMs and DEGs.

To better understand the regulatory relationship between genes and metabolites, we constructed a subnetwork regulatory map of the first 10 central genes and the first 10 central metabolites. The correlation analysis of transcriptome and metabolomics revealed a common metabolic pathway of DEGs and DAMs. The correlation between genes and metabolite was evaluated by Pearson’s coefficient of gene expression and metabolite abundance. Using the correlation coefficient, the top 205 DEGs and DAMs were selected. Metabolic pathways corresponding to (Figure 6) DAMs and DEGs indicated that DAMs and DEGs are largely involved in the ABC transporters process, followed by purine metabolism, glycine, serine and threonine metabolism, and alanine, aspartate and glutamate metabolism metabolic pathway. Among the top 10 hub genes, 3 were significantly enriched in the ABC transporters pathway, 2 were significantly enriched in the purine metabolic pathway, 2 were significantly enriched in the pentose phosphate pathway, and the other 3 were significantly enriched in the alanine, aspartate and glutamate metabolism, histidine metabolism, citrate cycle (TCA) metabolic pathway. In the top 10 metabolites, 5 DAMs participated in the ABC transporters pathway, whereas three DAMs participate in the ABC transporters and glycine, serine and threonine metabolism pathways. The two metabolites, M191T371_POS (L-Glutamine)and M146T392_2_NEG (Glutamic acid), participated in the alanine, aspartate, and glutamate metabolism, ABC transporters arginine biosynthesis, glyoxylate, and dicarboxylate metabolism at the same time. Among them, M191T371_POS(L-Glutamine) also participated in purine metabolism, and M146T392_2_NEG participated in histidine metabolic pathways.

### 3.6. NAA-mediated putative cordycepin metabolic pathway

**FIGURE 7:**
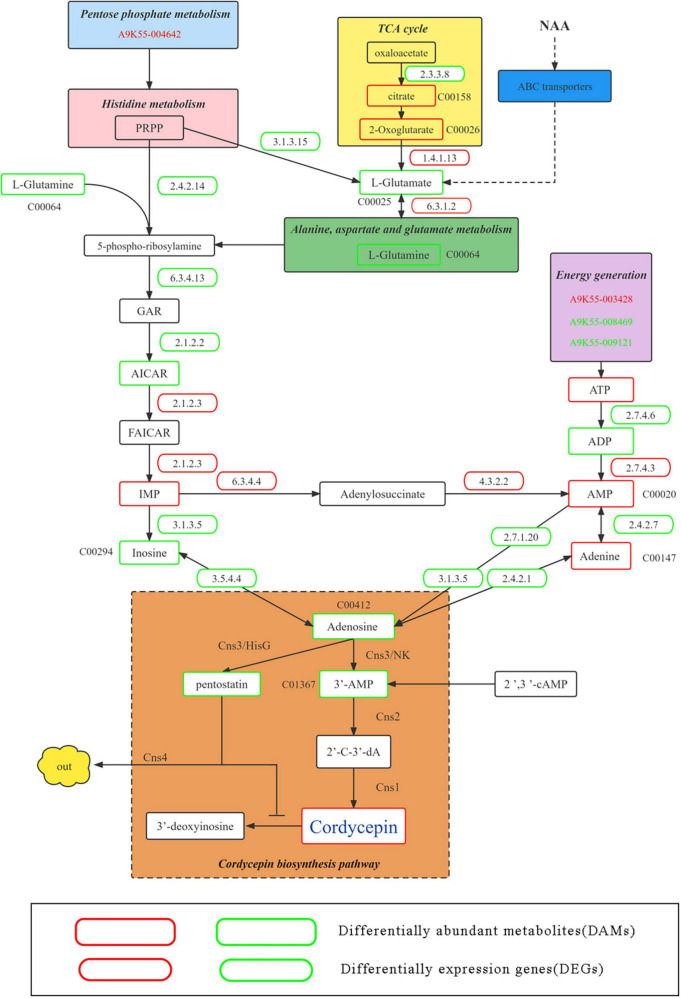
Putative pathway of cordycepin metabolism and synthesis. Green and red boxes represent downregulated and upregulated genes and metabolism and synthesis. Green and red gene numbers represent downregulated and upregulated genes, respectively.

Because the addition of NAA significantly improved the content of cordycepin, DEGs and DAMs corresponding to improved cordycepin content were identified in detail through the KEGG enrichment, hub gene screening, and gene and metabolite screening analysis. Therefore, we predicted the metabolic pathway related to cordycepin synthesis (Figure 7).

Previous studies have demonstrated the involvement of the pentose phosphate pathway and circular supply of citric acid in cordycepin biosynthesis as an energy supplier (35). At an NAA concentration of 4,000 mg/L, A9K55_004642 was commented as thermoresistant gluconokinase family proteins in the pentose phosphate pathway, (EC 2.7.1.12) which were significantly down-regulated (CK:18.36; N4000:9.7; multiple 0.53), causing significant down-regulation of L-glutamine content in purine metabolic pathways. Moreover, in citric acid circular, the upstream genes changed. For instance, A9K55_003137 (CK:5.97; N4000:2.61) and MSTRG.2470 (CK:160.04; N4000:106.6) were commented as ATP citrate (pro-S)-lyase (EC 2.3.3.8), which was significantly down-regulated, eventually causing significant up-regulation of 2-oxoglutarate content, a downstream product of the citric acid cycle. Because alanine, aspartate, and glutamate metabolism were affected by a significant increase in 2-oxoglutarate content in the citric acid cycle, the expression and levels of related genes and metabolites corresponding to the metabolic pathway changed accordingly. The transcription level of MSTRG.7321 (glt1) of DEGs was annotated as a glutamate synthase precursor (EC 1.4.1.13), which can convert 2-oxoglutarate to L-glutamate, which was significantly up-regulated (CK:62.3; N4000:97; multiple: 0.6). Under its influence, L-Glutamate and L-Glutamine were significantly downregulated. A9K55_000031 (SPCC1672.01) in the histidine metabolic belongs to the histidine alcohol phosphate phosphatase family (EC 3.3.3.15), which was significantly downregulated (ck: 3.75; N4000:1.3; multiple: 1.52), leading to significant downregulation of L-glutamate and that of L-glutamine in alanine, aspartate, and glutamate metabolic pathways. In addition, A9K55_008988 was noted as the amido phosphoribosyltransferase [EC 2.4.2.14]. Although its expression was downregulated, the difference was insignificant (CK:194.7; N4000:140; multiple: 0.4), and L-glutamine were downregulated to affect the downstream metabolic pathway of purine.

After the combined effect of the pentose phosphate pathway, alanine, aspartate, and glutamate metabolism, histidine metabolism and TCA cycle on purine metabolic pathways, the expression and levels of genes and metabolites were significantly altered. First, L-glutamine in the purine metabolism pathway was significantly downregulated, A9K55_000442 (ade5) corresponded to phosphoribosyl glycinamide formyl transferase (EC 2.1.2.2), which could convert GAR to AICAR and was significantly downregulated (CK: 253; N4000: 156.7; multiple 0.69). Although AICAR was down-regulated in control and experimental groups, no significant difference was found. A9K55_002919 (ADE17) was noted as bifunctional purine biosynthesis (EC 2.1.2.3), which could convert AICAR into FAICAR. Although no difference was observed in the transcription levels between the experimental and control groups, at an NAA concentration of 4,000 mg/L, the transcription level was the highest compared with other concentrations (CK:163; N4000:180.51), and its transcription level was higher than 46% of DEGs whose expression was more than 100. Although IMP was upregulated in the 4,000 mg/L group, each experimental group showed no significant difference. The transcriptional level of DEG A9K55_000049 was annotated as dechlorosuccinate synthase (EC 6.3.4.4), which converts IMP into adenosine succinate, showing upregulation (CK: 245; N4000:268; multiple 1.09). However, the difference was insignificant. For its downstream reaction, A9K55_003166 (EC 4.3.2.2) was used to generate AMP from adenylosuccinate, but did not show transcriptional differences between the samples. However, its transcription level was higher than 39% of DEGs with expression above 100 (CK: 198; N4000:200; multiple 1.01), and its upregulation significantly elevated the levels of AMP. In this study, seven genes were labeled as 1 5’-nucleotidase precursors (EC 3.1.3.5). However, no significant difference was noted between experimental and control groups. The transcription level of A9K55_006116 was 0.85 times, 0.85 times, and 0.90 times lower than that in the control group under each concentration. However, its expression was the highest among the seven genes, leading to the downregulation of inosine. A9K55_006221 is annotated as adenosine kinase (EC 2.7.1.20), which participates in the conversion between adenosine and AMP. Although its transcription level was relatively high (CK:110.56; N4000:118.49), no significant difference was noted between samples, and its downregulation significantly reduced the levels of adenosine. Two genes were annotated as adenosine deaminase (EC 3.5.4.4), A9K55_007735 and A9K55_002334, and showed a downregulation trend. The expression was low with no significant difference among experimental groups. Its downregulation caused a significant reduction in the levels of adenosine.

Energy metabolism has been implicated in the synthesis of cordycepin. The A9K55_003428 gene was annotated as a pyruvate kinase and showed an upward trend. However, we did not find any significant difference between samples, with a higher transcription level (CK:254; N4000:269). A9K55_008469 was annotated as ATP synthase 9 and A9K55_009121 was noted as a plasma membrane ATPase, showing a downward trend. However, no significant difference was noted between the samples. These three genes are responsible for upregulating the ATP. A9K55_007777 was annotated as a nucleoside diphosphate kinase (EC 2.7.4.6), which could convert ATP into ADP. Its downregulation (CK: 292; N4000:288) reduced the levels of ADP. A9K55_003828 was annotated as adenylate kinase (EC 2.7.4.3), which could convert ADP into AMP and was upregulated (CK:6.92; N4000:7.7), thereby significantly elevating AMP content. A9K55_007511 was annotated as an adenine phosphoribosyl transferase 1 (EC 2.4.2.7), which could convert AMP into adenine. The downregulation and upregulation of AMP caused upregulation of adenine content. A9K55_000590 was annotated as a purine nucleoside phosphorylase (EC 2.4.2.1), which could convert adenine into adenosine. Moreover, its downregulation significantly reduced the levels of adenosine. Therefore, adenosine was significantly downregulated under the effect of related genes and metabolites in the above pathways, and 0.6 times lower than the control group.

This network was connected to the core network of cordycepin synthesis. The four key genes, namely, Cns1, Cns2, Cns3, and Cns4, involved in cordycepin synthesis were analyzed. Cns1 was upregulated in the group treated with 4,000 mg/L NAA. However, it was downregulated in the other groups. Cns2 and Cns3 were upregulated in all groups. The dynamic changes in the expression of Cns1, Cns2, and Cns3 in different experimental groups indicated that the response of Cns1, Cns2, and Cns3 genes to NAA was different. The expression of *Cns1* was upregulated, indicating that at 4,000 mg/L NAA, the pathway of 2’-C-3’-dA converting to cordycepin was largely activated. In addition, upregulation of Cns2 and Cns3 indicated that adenosine was transformed into 3’-AMP, and massive 3’-AMP was transformed into 2’-C-3’-dA synthesis. Moreover, 3’-AMP was significantly downregulated. The high expression of Cns3 is conducive to the generation of PTN; however, compared with the control group, the content of PTN was reduced, which is consistent with the inhibition effect of Cns4 on the release of ADA by expelling PTN, which could help COR transform into 3’-deoxyinosine, and protect the cells from toxicity caused by high cordycepin content. Additionally, under four different NAA concentrations, 13 DEGs (13/2353, 0.5%) corresponding to ATP-binding cassette (ABC) transporters were involved in metabolism. Sixty-one DAMs (61/924, 6.6%) were found enriched in the ABC transporter pathway (Supplementary Table 6). According to the statistics, most ABC transporters transport amino acids and sugars to participate in NAA transformation and cordycepin metabolism and improve the production of cordycepin. For instance, L-glutamate was significantly downregulated in the 4,000 mg/L NAA group, inferring that L-glutamate participates in the synthesis of cordycepin, connecting the metabolic pathways of alanine, aspartate, and glutamate metabolism, pentose phosphate pathway, and TCA cycle. It further affected the purine metabolic pathway through the histidine metabolic pathway, significantly increasing the content of cordycepin at 4,000 mg/L NAA. The expression of related significant DEGs and the accumulation level of related significant DAMs in the metabolic pathway is shown in Supplementary Figure 6.

### 3.7. Validation of gene expression patterns by qRT-PCR

**FIGURE 8:**
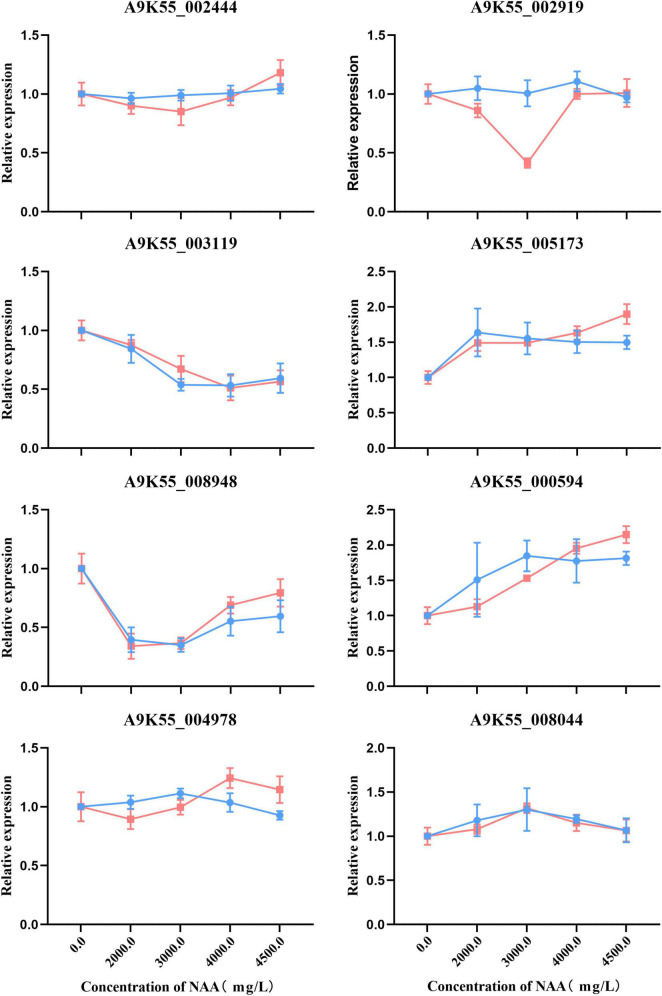
Real-time quantitative polymerase chain reaction (RT-qPCR) validation of eight genes. Expression patterns of the eight genes involved in purine metabolism, fatty acid metabolism, amino sugar and nucleotide sugar metabolism, glycine, serine, threonine metabolism, and other genes on the metabolic pathway. Each column represents an average of three biological replicates, with standard errors indicated by vertical bars. Values with a different accompanying letter are statistically significantly different according to Duncan’s multiple range test at *p* < 0.05.

We used qRT-PCR to verify that the *C. militaris* fruiting body treated with different concentrations of NAA was involved in purine metabolism, fatty acid metabolism, amino sugar and nucleotide sugar metabolism, glycine, serine, threonine metabolism, and other genes of the metabolic pathway. The gene expression correlation between qRT-PCR (i.e., relative expression) and RNA-seq data (i.e., FPKM value) of *C. militaris* fruiting body treated with different concentrations of NAA was consistent (Figure 8).

## 4. Discussion

Previous studies have obtained high yields of cordycepin by optimizing the composition of the medium and changing culturing conditions. However, the addition of different substances and improvement in culturing conditions were performed using the *C. militaris* mycelium stage (11, 14). The literature on the culture stage of *C. militaris* fruiting body is scarce. Because the fruiting body of *C. militaris* has emerged as a new resource of food raw material, there is an industrial need to cultivate a high cordycepin fruiting body. Yuan et al. studied the addition of NAA to improve the content of cordycepin in *C. militaris* fruiting bodies; however, the underlying molecular mechanism remains unclear (22). To better understand the adaptation of *C. militaris* to NAA stress conditions, we conducted relevant physiological experiments to explore the impact of NAA treatment on cordycepin. The addition of high-concentration NAA creates a stressful environment for *C. militaris* and correspondingly prolongs the time of occurrence of bacteria during the dark culture stage. This finding is consistent with that of a previous study that reports that NAA delays the growth rate of mycelia (36). At the reproductive growth stage, different concentrations of NAA significantly inhibited the growth of *C. militaris* fruiting body. With an increase in the NAA concentration, the dry weight of *C. militaris* fruiting body showed a significant downward trend (*P* < 0.05). The content of cordycepin was significantly improved (*P* < 0.05). At 4,000 mg/L NAA, the content of cordycepin reached the maximum of 675.9 mg/100 g, which was 4.6 times the cordycepin content of the control group. At 4,500 mg/L NAA, the cordycepin content decreased, whereas at 5,000 mg/L NAA, the fruiting body did not grow. Hence, the addition of NAA changes the permeability of the cell membrane, causing certain toxicity to *C. militaris*, disrupting their normal physiological metabolism, and inhibiting their growth. An increase in NAA concentration increases the accumulation of toxic substances caused by serious damage to the cell membrane, reducing the ability to grow. During stress, the body undergoes major changes in morphology, and primary and secondary metabolites to adapt to the environment for survival, which is consistent with the corresponding physiological characteristics of *Saccharomyces cerevisiae*, *Streptomyces caeruleus*, and *C. militaris* (high salt stress) under stress conditions (37–39). The fruiting body undergoes different morphological, physiological, and molecular changes to adapt to the environment. Therefore, the change in the external phenotype of *C. militaris* could be attributed to the dynamic changes in the expression of related genes after adding a high concentration of NAA.

The WGCNA method was used to understand the correlation between gene expression on the metabolic pathway related to cordycepin synthesis and screen hub genes. Hub genes were enriched in purine metabolism and sphingolipid metabolic pathway, lipid metabolic pathway, phosphoinositide metabolism, phosphoinositide metabolic pathway, inositol phosphate metabolism, ether lipid metabolism, histidine metabolism, cyanoamino acid metabolism, glycine, serine, and threonine metabolism, cysteine, and methionine metabolism, ABC transporters, and starch and sucrose metabolism. We reported a enrichment of sphingolipid metabolism, fatty acid metabolism, glycerophospholipid metabolism, inositol phosphate metabolism, ether lipid metabolism following treatment with a high concentration NAA. This finding indicated that treatment with high concentrations of NAA changed the membrane composition and structure, altered its permeability, and disrupted its corresponding physiological functions (40, 41). Studies have implicated the sphingolipid metabolic pathway in cell growth and antistress reaction. The significant enrichment of this pathway showed that cell growth is affected by adverse stress, which is consistent with the significant decline noted in the dry weight of *C. militaris* with an increase in NAA concentration (42). Under high salt conditions, glyceride metabolism and high osmotic glycerol (HOG) signal pathways in *S. cerevisiae* enable microorganisms to respond to different extracellular stimuli and adjust their cellular mechanisms to alter their environment (39, 43). The *C. militaris* strain significantly enriches the biosynthesis pathway and glyceride metabolism pathway of unsaturated fatty acids under high salt conditions. Therefore, the reaction of *C. militaris* under NAA treatment is similar to that of *S. cerevisiae* and *C. militaris* under a high salt environment (39). As an important component of the cell membrane, lipids are highly sensitive to environmental changes. Under external stress, the corresponding lipid-related metabolic pathway changes are consistent with the research results in this study (44). Furthermore, metabolic pathways of amino acids such as histidine, cyanoamino acid, glycine, serine and threonine, and cysteine and methionine are significantly enriched; thus, we speculated that cordycepin synthesis is related to amino acid metabolism. Chen et al. reported an increased production of cordycepin by adding L-alanine to activate the mutual transformation of amino acids. Cysteine, glutamic acid, glutamine, glycine, and aspartic acid are involved in mutual transformation, directly stimulating the metabolite transformation of histidine metabolism, and indirectly participating in the cordycepin synthesis pathway (45). Wongsa et al. proved that glycine and L-glutamic acid could be used as a precursor pool of cordycepin biosynthesis (13). Raethong et al. developed a regulatory network of adenosine, methionine, and cordycepin (25). Xia et al. reported a three-gene biosynthesis cluster for cordycepin synthesis using adenosine, indicating that cordycepin metabolism partially overlaps with the purine pathway. In this study, hub genes were significantly enriched in the purine metabolic pathway (24). Therefore, we can conclude that amino acid metabolism, purine metabolism, and cordycepin synthesis are intricately related, among which the histidine metabolic pathway has been implicated in the synthesis of cordycepin as a bypass pathway of purine metabolism. In addition, the starch and sucrose metabolic pathways were significantly enriched, consistent with the results of a previous study (45). Moreover, hub genes were significantly expressed in the ABC transporter pathway. Xia et al. demonstrated that Cns4 belongs to the ABC transporter family; however, it only transports PTN. In this study, A9K55_008246 did not belong to Cns4, indicating that ABC transporters also participate in the transport of other metabolites, thereby promoting cordycepin synthesis. Studies have demonstrated that most ABC transporters have transport activity in the organism and rely on the energy generated by ATP hydrolysis for the transmembrane transport of substrates inside and outside the cells. Their transport substrates include polysaccharides, amino acids, polypeptides, heavy metal chelates, alkaloids, and drugs (46–48). As reported, ABC transporters are involved in wax secretion on plant surface, regulation of heavy metals, secondary metabolites transportation, etc., (49).

Metabolomics data show that the number of DAMs reached the maximum at 4,000 mg/L NAA. It can be speculated that 4,000 mg/L is the most appropriate concentration of NAA for *C. militaris*. To further explore the specific categories of DAMs and the involved metabolic pathway, we performed KEGG enrichment analysis on DAMs. The results showed that, compared with the control group, at NAA concentrations of 2,000, 3,000, and 4,000 mg/L, and DAMs were largely concentrated in amino acid metabolism, carbohydrate metabolism, and nucleotide metabolism, respectively, whereas at an NAA concentration of 4,500 mg/L, DAMs were mostly concentrated in amino acid metabolism, carbohydrate metabolism, and metabolism of other amino acids. Therefore, the addition of NAA affected the metabolism of amino acids and carbohydrates, thereby affecting cordycepin synthesis. In addition, naphthalenes were detected in the metabolomics data, indicating that NAA exists in the form of 1-naphthalene acetamide and naphthol. With an increase in the concentration, the content of NAA increased first and subsequently decreased. At 4,000 mg/L, the content of NAA reached the maximum, whereas the overall content of naphthol showed a downward trend. NAA may provide amino groups for the synthesis of cordycepin. Adenosine, adenine, ADP, AMP, hypoxanthine, pentostatin, and cordycepin were detected in the purine metabolic pathway. At an NAA concentration of 4,000 mg/L, the ADP content reached the minimum and the AMP content reached the maximum. In this case, the adenine content and the cordycepin content reached the maximum, whereas the adenosine content was the minimum. Thus, we speculate that adenosine is used for large-scale synthesis of cordycepin, resulting in the lowest adenosine content. This is consistent with the idea that adenosine is used as a precursor for cordycepin synthesis, as demonstrated by Xia et al. (24). At a certain level of cordycepin, it can be deaminated by adenosine deaminase to produce non-toxic 3-deoxyinosine, whereas PTN can prevent cordycepin deamination by inhibiting the activity of adenosine deaminase. At 4,000 mg/L NAA, the content of PTN decreased significantly compared with the control group, which is consistent with that reported in previous studies (24). Furthermore, PTN can be produced through adenosine. The adenosine content reached the minimum value at this time, whereas cordycepin content reached the maximum value. Adenine, xanthine, and hypoxanthine were significantly upregulated, suggesting that the upregulation of xanthine can inhibit the generation of IMP from AMP *via* a feedback mechanism. AMP can be hydrolyzed into adenosine, which can be converted into hypoxanthine, and hypoxanthine can be converted into xanthine. Upregulation of xanthine can inhibit the generation of IMP from AMP *via* a feedback mechanism.

The regulatory relationship between genes and metabolites showed that hub genes and metabolite largely participate in the following metabolic pathways: purine metabolism, phosphoinositide metabolic pathway, histidine metabolism, glycine, serine and threonine metabolism, alanine, aspartate and glutamate metabolism, ABC transporters, pentose phosphate pathway, starch and sucrose metabolism, cyanoamino acid metabolism, TCA cycle, and other metabolic pathways, which is also consistent with the previous transcriptome data. Thus, the addition of NAA changes the permeability of the cell membrane, increases the production of energy molecules, and correspondingly affects the metabolism of amino acids, further improving the production of cordycepin.

It has been previously reported that the synthetic pathway of cordycepin is primarily the purine metabolic pathway. However, how it is regulated and how it functions with other metabolic pathways remain unclear. We conducted a comprehensive analysis based on the relevant metabolic pathways identified by previous researchers and the results of this study and drew a sketch of the multi-pathway synergy of cordycepin synthesis. The treatment with NAA significantly enriched ABC transporter metabolic pathways. The ABC transporter plays an important role in the transmembrane transport of highly compatible organics (50). The NAA related transport was performed, thus initiating the expression of certain related genes. Combined with metabolomics data, L-glutamine was transported in large quantities and was significantly downregulated. Certain genes corresponding to ABC transporters (Supplementary Table 6) could be involved in the inter-transformation of amino acids and cordycepin metabolism *in vivo*, which is consistent with a previous study that reported that L-glutamine can significantly promote cordycepin production (51). On the one hand, L-glutamine promoted the mutual transformation between alanine, aspartate, and glutamate metabolism, pentose phosphate pathway, and TCA cycle, further affecting the histidine metabolic pathway, as a bypass route of purine metabolism, through A9K55_000442 (ade5) on the histidine metabolic pathway, which is noted as a significant downregulation of phosphoribosyl glycinamide formyl transferase (E2.1.2.2) and L-glutamine, affecting the conversion of PRPP to AICAR, and thus impacting the production of IMP. Although the change in IMP is insignificant, it was upregulated at 4,000 mg/L NAA compared with the control group. Adenyl succinate synthase (EC 6.3.4.4) A9K55_000049 can convert IMP to adenosine succinate, and afterward, adenosine succinate lyase (EC 4.3.2.2) A9K55_003166 catalyzes the reaction producing AMP. Although both these genes are expressed, no significant difference between the transcripts was observed. Its transcription level was higher than other DEGs, and the AMP content was significantly increased. The ADP content reached the minimum. There is substrate feedback inhibition that reduces the ADP content. Changes in the expression of these genes and metabolites increased the content of cordycepin.

On the other hand, the relationship between genes and genes, genes and metabolite implicated energy metabolism in the synthesis of cordycepin. Pyruvate kinase A9K55_003428 transcript is expressed at a high level, which is conducive to ATP generation and is consistent with the finding of a previous study (45). A9K55_008469 and A9K55_009121 were downregulated. The inhibition of the expression of H^+^-ATPase genes is conducive to the accumulation of ATP (52); therefore, the above genes participate in the cordycepin synthesis through ATP. The production of adenosine is affected by inosine, AMP, and adenine. AMP and adenine content is significantly increased, whereas the inosine content was significantly decreased. It is speculated that there could be a high content of AMP. After adenine is transformed into adenosine, it is immediately transformed into 3’-AMP and pentostatin under the action of cns3. The content of 3’-AMP is lower than that in the control group. It is speculated that 2’-C-3-dA is immediately synthesized under the action of cns2. The content of pentostatin was lower than that of the control group, implying it is transported out of the cell. Thus, adenosine deaminase contributes to the deamination of cordycepin to generate non-toxic 3’-deoxyinosine. Therefore, adenosine content was the lowest, and cordycepin content was the highest. The content added by a large concentration of NAA but ultimately left in the fruiting body was highly small. Whether NAA is used as a substrate to participate in the metabolism or as a catalyst to affect the activity of related enzymes to improve the cordycepin content requires further verification. Under a high-stress environment, multiple metabolic pathways are affected. There is a synergy between metabolic pathways, improving the cordycepin content. Cordycepin is only one of the effective substances, and other effective substances can be further identified. We believe that this study will guide the production to obtain high levels of cordycepin fruiting body, and provide a reference for further cordycepin biosynthesis and gene editing.

## 5. Conclusion

Based on the results of previous studies and this study, we elucidated the metabolism network diagram that the addition of NAA improves the content of cordycepin. The synthesis of cordycepin is affected by multiple pathways. Treatment with NAA activates several metabolic processes and pathways including the TCA cycle, pentose phosphate pathway, amino acid metabolism, energy metabolism, and ABC transporter pathway. TCA cycle, pentose phosphate pathway, and amino acids metabolic pathway are connected through L-glutamate. Finally, NAA indirectly promotes the content of IMP *via* the histidine metabolic pathway. The changes in the levels of AMP, inosine, adenine, pentostatin, and 3’-AMP content resulted in the production of activated enzymes related to adenosine production, thereby increasing the levels of adenosine-producing cordycepin. Furthermore, the ABC transporter pathway promoted the synthesis of cordycepin. Numerous amino acids such as L-glutamate are transported, playing a role in amino acid metabolic pathways and indirectly promoting the synthesis of cordycepin. Thus, we proved the hypothesis that cordycepin synthesis is synergized by multiple metabolic pathways in addition to the purine metabolic pathway.

## Data availability statement

The data presented in this study are deposited in the NCBI, accession number PRJNA900787 (https://www.ncbi.nlm.nih.gov/bioproject/PRJNA900787).

## Author contributions

XW and XC: conceptualization. XL: methodology. LS: software. YF, XL, and FS: validation. YuL: formal analysis. SY: investigation. YG: resources. XW: data curation, writing – original draft preparation, visualization, and project administration. XC and WL: writing – review and editing. YiL: supervision. All authors have read and agreed to the published version of the manuscript.

## Footnotes

1
http://www.r-project.org/
