# Supplementary material for: Transcriptome and metabolome profiling unveils the mechanisms of naphthalene acetic acid in promoting cordycepin synthesis in Cordyceps militaris
Source: Front Nutr. 2023 Feb 16;10:1104446. doi: 10.3389/fnut.2023.1104446 (PMC9977999; doi:10.3389/fnut.2023.1104446)
Supplement: Supplementary file 1 [file Presentation_1.pdf]

Supplementary Material

Supplementary Figures

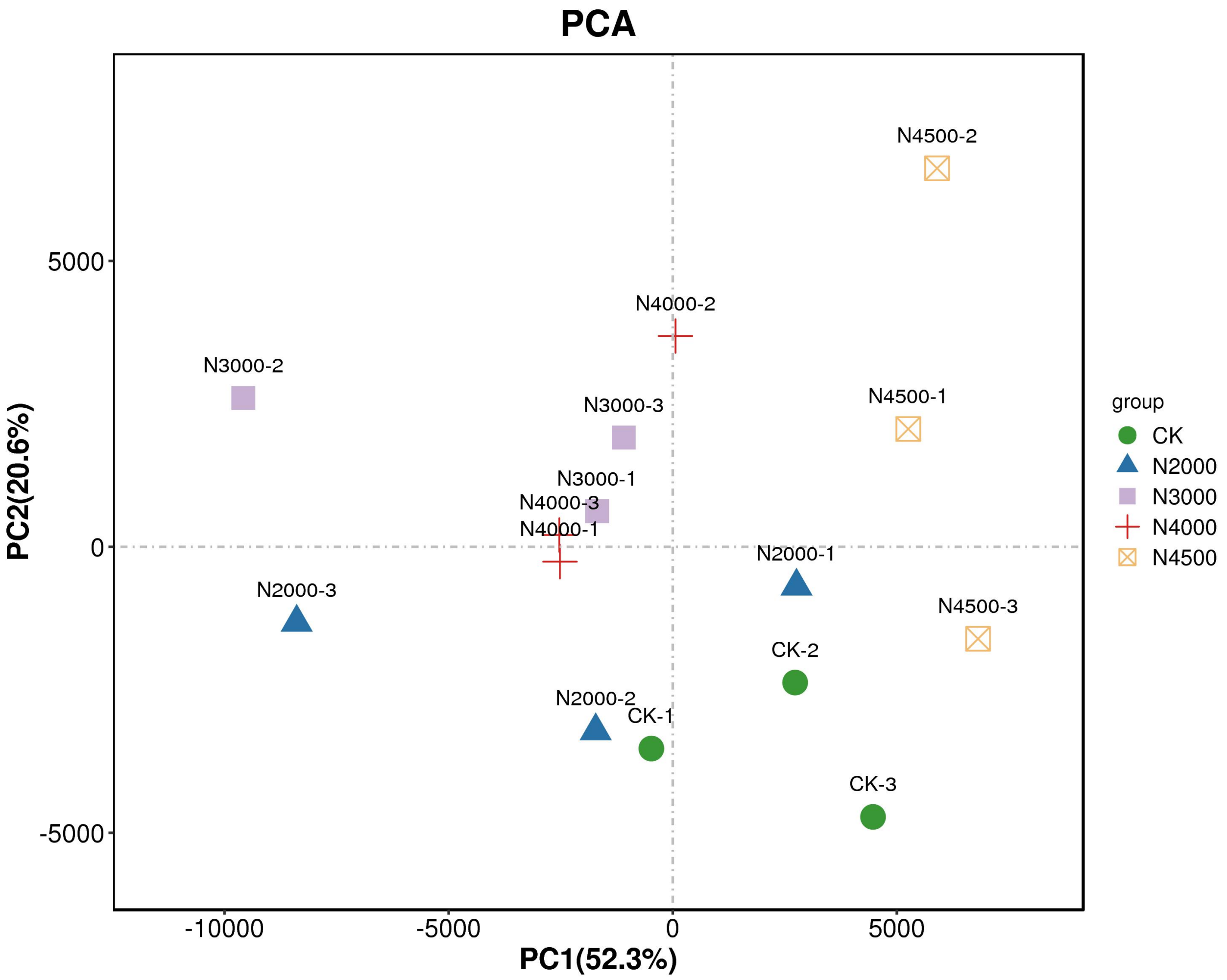

Supplementary Figure 1. PCA analysis of control and different treatment samples.

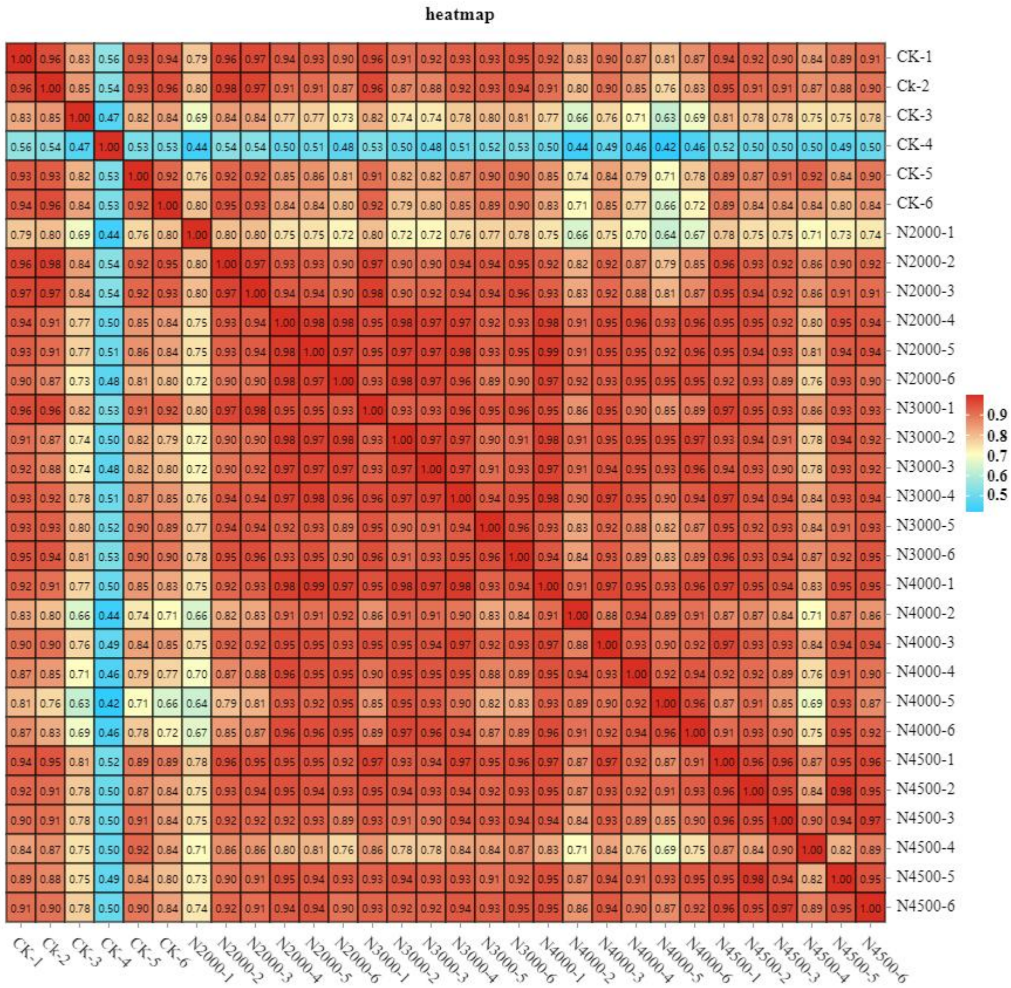

Supplementary Figure 2. A heatmap with pearson correlation analysis of control and different treatment samples.

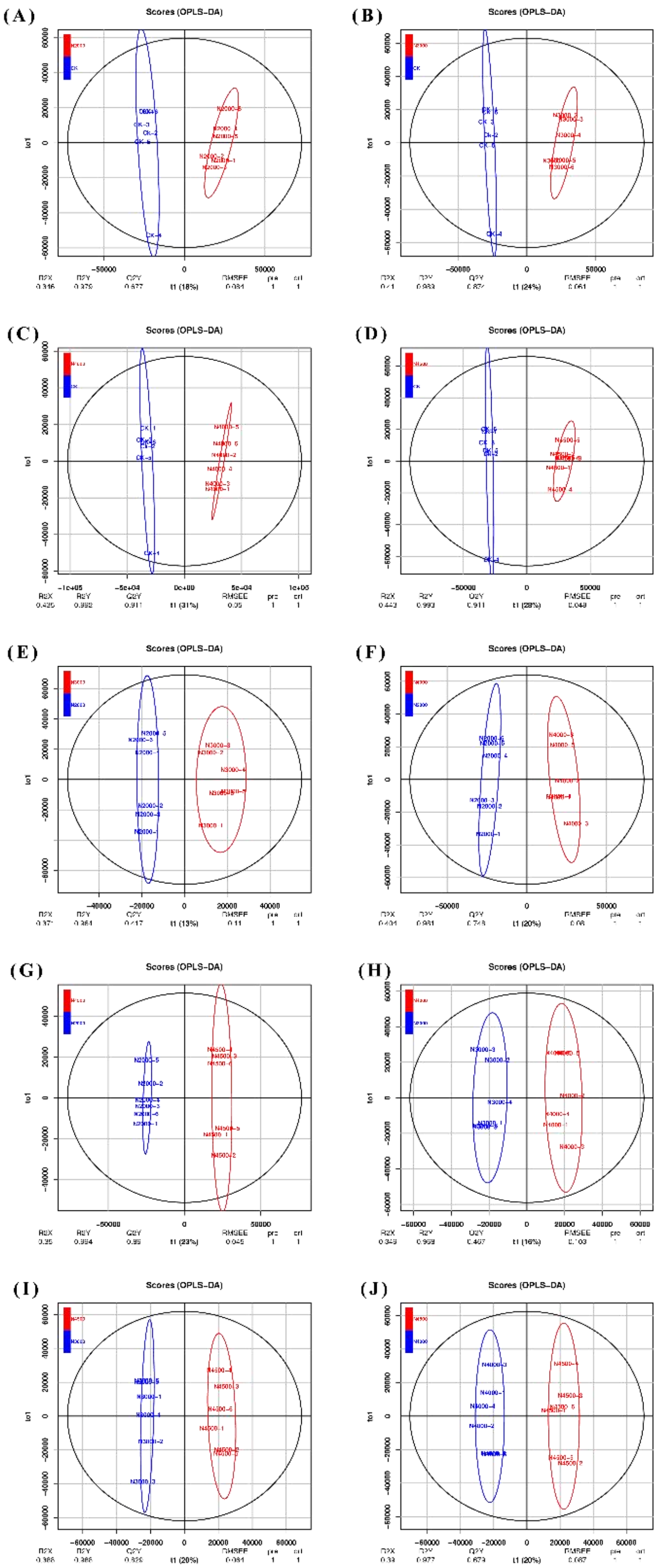

Supplementary Figure 3. Plots from orthogonal projection to latent structures-discriminant analysis (OPLS-DA) for modeling the differences between two comparison groups (A)CK and 2000mg/L (B) CK and 3000mg/L (C) CK and 4000mg/L (D) CK and 4500mg/L (E) 2000mg/L and 3000mg/L (F)

2000mg/L and 4000mg/L (G)2000mg/L and 4500mg/L (H) 3000mg/L and 4000mg/L (I) 3000mg/L and 4500mg/L (J) 4000mg/L and 4500mg/L;

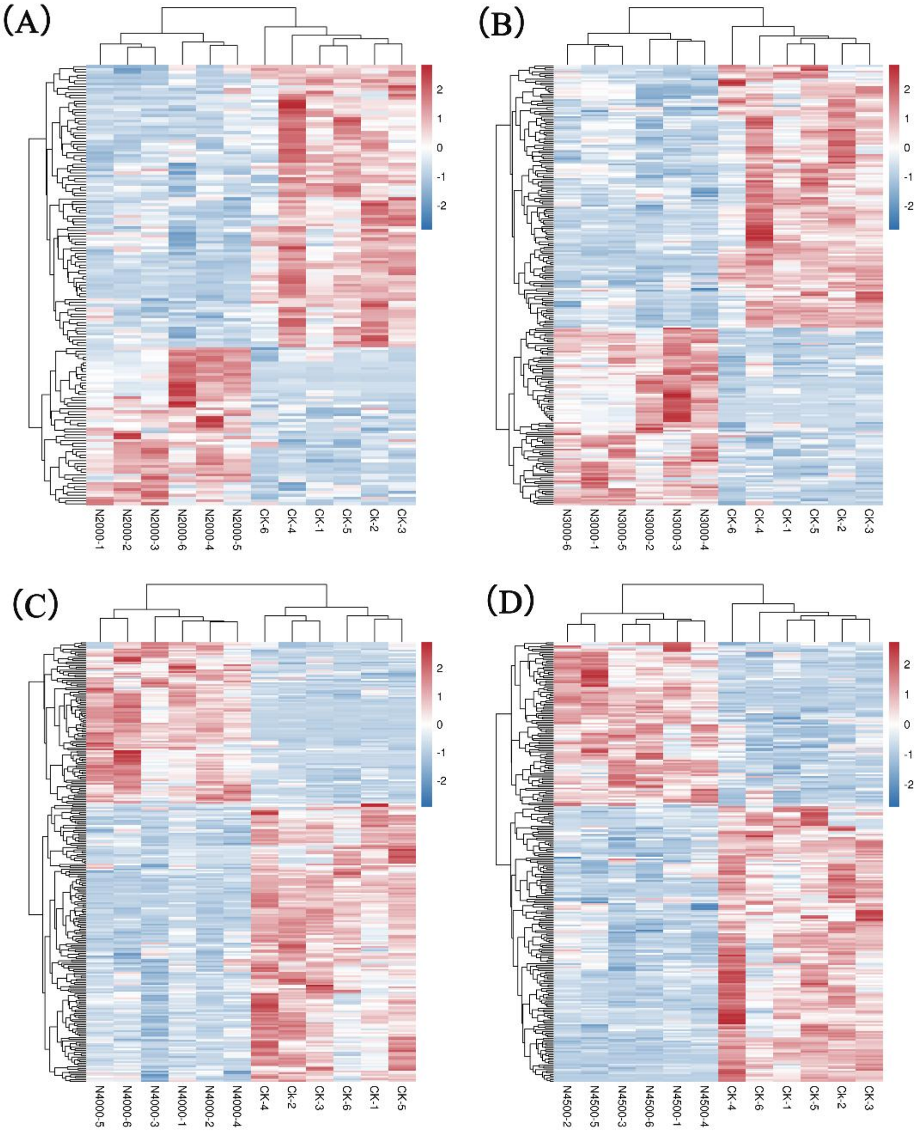

All Diff Metabolite Heatmap

Supplementary Figure 4. Heatmap of differentially accumulated metabolites (DAMs) between CK and other groups (2000mg/L、3000mg/L、4000mg/L、4500mg/L) in *C. militaris.* (A) Heatmap representing the hi-erarchical cluster analysis in CK vs. 2000mg/L. (B) Heatmap representing the hierarchical cluster analysis in CK vs. 3000mg/L. (C) Heatmap representing the hierarchical cluster

analysis in CK vs. 4000mg/L. (D) Heatmap representing the hierarchical cluster analysis in CK vs. 4500mg/L. The colors from blue to red indicates low to high accumulation.

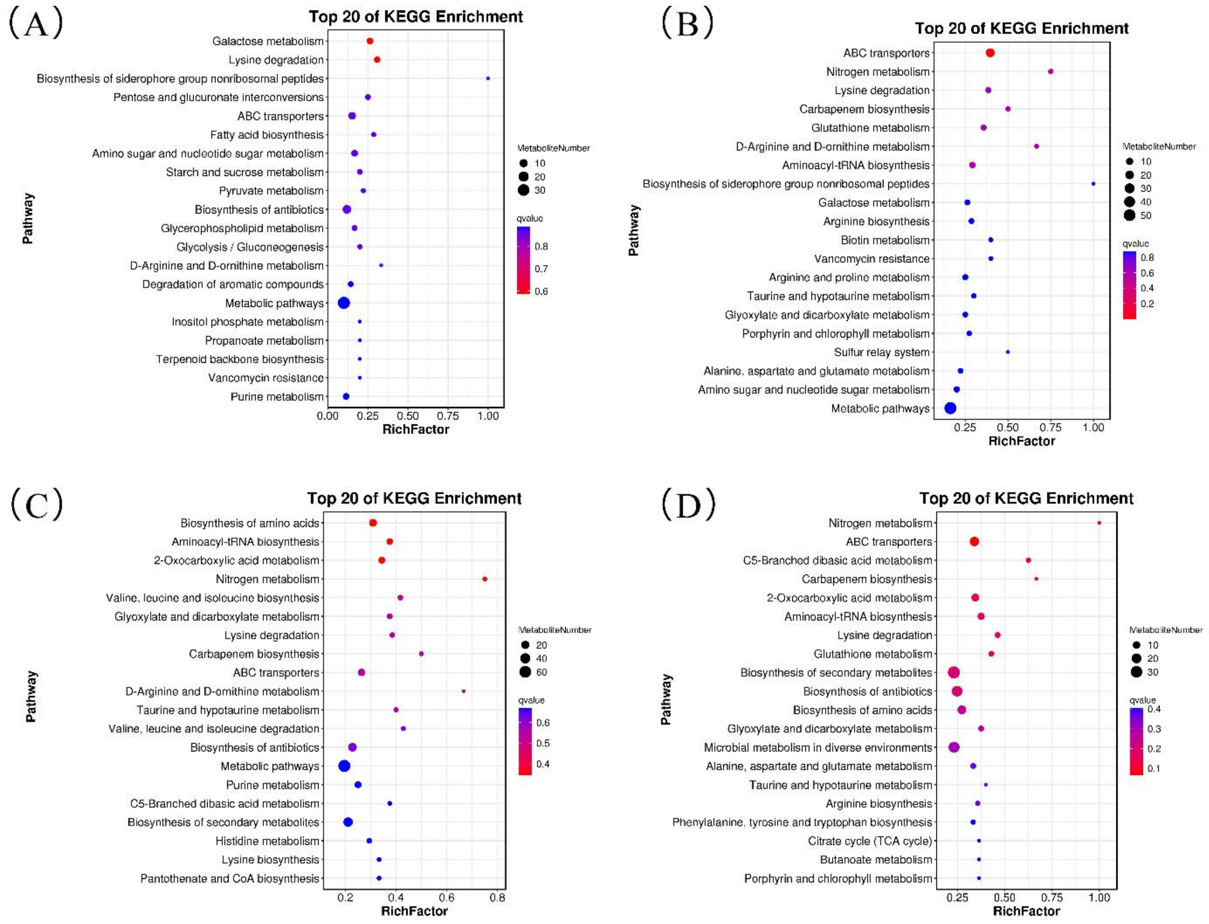

Supplementary Figure 5. KEGG pathway enrichment analysis of DAMs. (A) Pathway enrichment in CK vs. 2000mg/L. (B) Pathway enrichment in CK vs. 3000mg/L. (C) Pathway enrichment in CK vs. 4000mg/L. (D) Pathway enrichment in CK vs. 4500mg/L. The x-axis represents the enrichment factor, while the y-axis represents the enrichment pathway. The dot sizes represent the number of DAMs. The statistical analysis of the pathway enrichment was performed using Fisher's exact test. A lower q-value indicates that a lower percentage of significant results will be false positives. Rich factor represents the degree of enrichment of genes under the designated pathway term. Greater the value of the rich factor, greater is the degree of pathway enrichment.

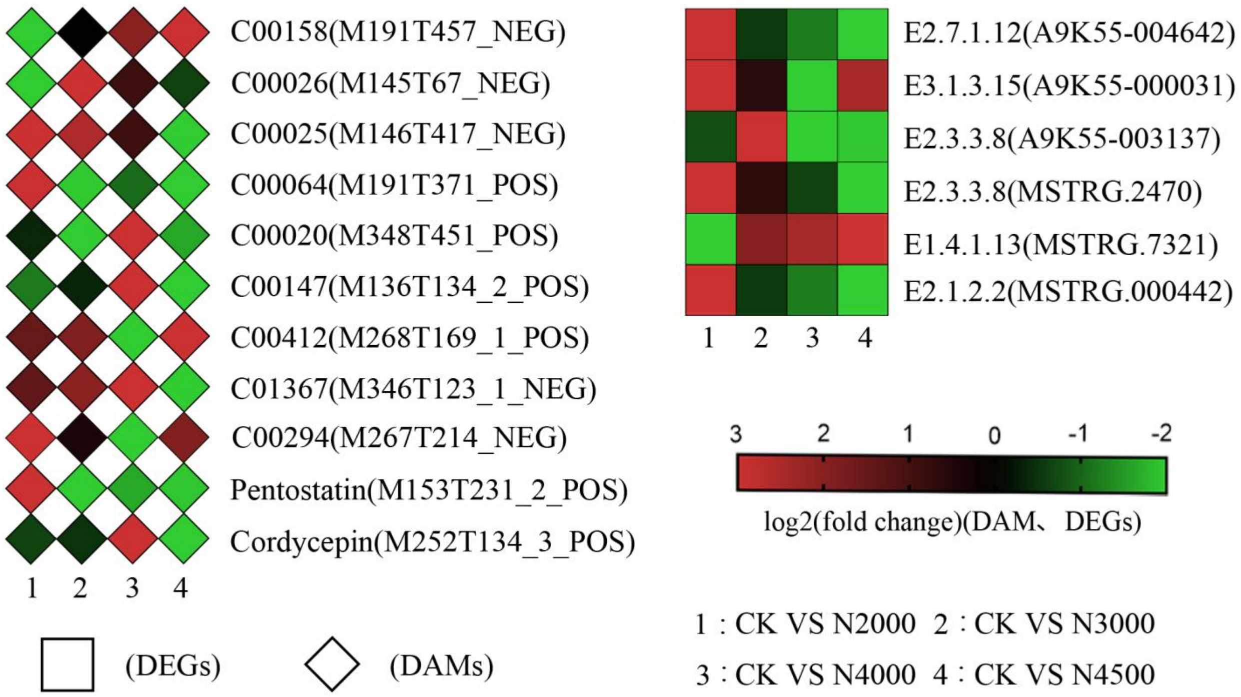

Supplementary Figure 6. The expression of related significant DEGs and the accumulation level of related significant DAMs in the metabolic pathway.
